# Factors Associated With End‐of‐Life Care Competencies Among Nurses in Departments With a High Incidence of End‐of‐Life Cases: A Mixed‐Methods Study

**DOI:** 10.1155/jonm/8236573

**Published:** 2026-07-15

**Authors:** So Hyeon Yeon, Kyung Im Kang

**Affiliations:** ^1^ Hospice Center, Gyeongsang National University Hospital, Jinju, South Korea, gnuh.co.kr; ^2^ College of Nursing, Sustainable Health Research Institute, Gyeongsang National University, Jinju, South Korea, gnu.ac.kr

**Keywords:** end-of-life care competency, ethical nursing competency, mixed methods, perception of shared decision-making, positive psychological capital, self-leadership

## Abstract

**Background:**

Recent medical advances have led to longer lives, shifting end‐of‐life care from families to healthcare institutions. Despite the critical need to identify factors influencing end‐of‐life care competency among nurses in high‐mortality departments, this area remains underexplored.

**Aim:**

This study aimed to determine the factors associated with end‐of‐life care competency among nurses in high‐mortality departments, with a focus on preserving patient dignity and quality of life.

**Design:**

An explanatory sequential mixed‐methods design.

**Methods:**

A survey was administered to 204 nurses across three general hospitals in G City, Korea, from June 13 to July 11, 2024, and data were analyzed using hierarchical regression. Subsequently, focus group interviews were conducted with nine participants between August 21 and October 10, 2024, followed by thematic analysis.

**Results:**

Quantitative analysis revealed that the perception of shared decision‐making (*β* = 0.20, *p* = 0.002), ethical nursing competency (*β* = 0.31, *p* < 0.001), and positive psychological capital (*β* = 0.19, *p* = 0.030) significantly influenced end‐of‐life care competency among nurses working in high‐mortality departments. The regression model demonstrated an explanatory power of 46.8% (*F* = 14.04, *p* < 0.001, *R* ^2^ = 0.468, Adj‐*R* ^2^ = 0.435). Thematic analysis yielded five themes and twelve subthemes.

**Conclusion:**

This study identifies key factors to enhance end‐of‐life care competency among nurses in high‐mortality departments. The findings provide specific evidence to improve care quality and optimize nurse deployment in high‐demand settings.

**Implications for Nursing Management and Leadership:**

This study highlights important implications for nursing leadership in improving end‐of‐life care. Nurse managers play a key role by providing structured training and fostering an ethical, supportive organizational climate. They must also promote effective communication and shared decision‐making within clinical teams. Especially in high‐mortality settings, leaders should prioritize workforce development strategies that strengthen nurses’ psychological resilience and reduce burnout.

## 1. Introduction

Contemporary developments in medicine are contributing to increased life expectancy, prompting a shift in end‐of‐life care from the home to healthcare facilities and in primary caregiving from family members to healthcare providers [1]. According to Statistics Korea, the proportion of deaths occurring in healthcare facilities increased from 70.1% in 2012 to 74.8% in 2022, and the leading causes of death have been cancer, pneumonia, and cardiocerebrovascular diseases over the past five years [2]. Patients with these conditions typically receive care in intensive care units (ICUs), emergency departments, oncology units, cardiology units, and respiratory care units. Nurses working in these departments are more likely to have end‐of‐life care experiences than those in other departments [3–5].

This growing trend in hospital‐based mortality highlights the expanding role of nurses in end‐of‐life care delivery. As more patients die in medical settings, the frequency and intensity of end‐of‐life care provided by nurses will continue to increase. End‐of‐life care refers to supportive care provided by nurses to patients who are in the process of dying and to their families [6]. Nurses play a critical role in providing patient‐centered care to help patients navigate this process [7]. However, nurses who regularly care for dying patients often experience several challenges including psychological burden and burnout. These challenges can negatively affect the nurse–patient relationship, reduce the quality of care, and lower job satisfaction [8]. Such challenges may arise when nurses are required to consistently deliver end‐of‐life care despite lacking adequate competence [9]. Therefore, identifying relevant influencing factors and strengthening nurses’ competencies are essential for improving the quality of end‐of‐life care.

To understand the multidimensional nature of these competencies comprehensively, Hökkä et al. [10] proposed an evidence‐based conceptual framework of nursing competencies in palliative care based on a systematic integrative review with thematic synthesis. This framework encompasses six domains: collaboration with patients and families, communication and cultural competence, clinical competence, ethical–legal competence, psychosocial and spiritual competence, and professional role and leadership. Although this conceptual framework spans these six competence domains, an integrative consideration of its components alongside additional empirical evidence [4, 11–16] confirms that shared decision‐making, ethical nursing competency, self‐leadership, and positive psychological capital are significantly associated with end‐of‐life care competency among nurses.

Aligned with the domains of collaboration and communication, nurses caring for patients at the end of life should be knowledgeable about shared decision‐making to improve the quality of patient‐centered care and end‐of‐life care. Increasing attention has been given to the need for clear guidelines, education, and training on shared decision‐making, as research shows that it improves satisfaction among physicians, caregivers, and patients [17]. In Korea, medical decisions at the end of life are often made between physicians and patients’ families, frequently excluding the patients themselves from the decision‐making process [18]. Despite its importance, the association between perceptions of shared decision‐making and the quality of end‐of‐life care remains understudied in Korea. Therefore, further research is needed to explore the relationship between these two variables.

Reflecting the ethical–legal domain, nurses often encounter various ethical conflicts when caring for patients at the end of life [19, 20]. To effectively address these challenges, they are expected to possess strong ethical competencies guided by professional codes of conduct. A low level of ethical competence can negatively affect nursing practice [21]. In addition, the vulnerability and limited decision‐making capacity of patients at the end of life must be carefully considered to uphold and advocate for their rights [11]. Therefore, ethical nursing competence is regarded as a critical factor in predicting the overall quality of nursing care in end‐of‐life situations.

Within the domain of professional role and leadership, the leadership role of nurses is also an essential factor in enhancing nurses’ end‐of‐life care competency. Effective leadership is fundamental to nursing practice, enabling nurses to respond competently to sudden changes in patients’ conditions and to act promptly and appropriately in clinical settings [10]. Self‐leadership, an intrinsic form of motivation and autonomy, contributes to improved job performance [22]. Studies have also shown that it positively influences job performance, well‐being, job satisfaction, and organizational commitment among nurses [12]. Given its established associations with various professional nursing outcomes, self‐leadership was considered a potentially relevant factor for end‐of‐life care competency. In this regard, it may facilitate end‐of‐life care competency by enhancing nurses’ self‐regulation, goal‐directed behavior, and reflective practice, which are essential for making autonomous and timely clinical decisions in complex care situations.

From a psychosocial perspective, nurses who care for patients at the end of life are highly susceptible to psychological burden and emotional exhaustion [8]. To overcome these psychological challenges, positive thinking and the ability to return to a stable emotional state, as components of positive psychological capital, are essential [23]. Positive psychological capital emphasizes individuals’ strengths and potential within an organization. Studies show that it positively influences physical and psychological health, work engagement, and job performance [4, 13, 24, 25]. In clinical settings, where nurses are expected to take a proactive role with a positive mindset, it is important to foster positive psychological capital through targeted education and training programs [26, 27]. Thus, the relationship between positive psychological capital and end‐of‐life care competency requires further investigation. In this regard, positive psychological capital may contribute to end‐of‐life care competency by enabling emotional regulation, resilience, and sustained engagement, thereby helping nurses maintain high‐quality care in emotionally demanding end‐of‐life situations.

Previous studies of end‐of‐life care competencies have primarily focused on nurses working in single departments, such as ICUs [28], oncology units [14], long‐term care hospitals [15], or tertiary hospitals [16]. Consequently, there is a lack of comprehensive research encompassing multiple departments in which end‐of‐life care frequently occurs. Therefore, it is necessary to conduct a study that examines end‐of‐life care competency among nurses in various high‐incidence departments, including ICUs, emergency rooms (ERs), oncology units, cardiology care units, and respiratory care units, as well as the factors associated with end‐of‐life care competencies.

Given the complex and multidimensional nature of end‐of‐life care competency, a single quantitative approach may be insufficient to fully capture how these competencies are understood and enacted in clinical practice. While quantitative analysis can identify relationships among key variables, it has limitations in explaining the contextual and experiential aspects underlying these relationships [29]. Therefore, a qualitative phase was included to provide in‐depth explanations of these findings within real clinical contexts [29].

Accordingly, this study adopted an explanatory sequential mixed‐methods design, following Creswell’s approach [29], to further explain and interpret quantitative findings through qualitative data. This approach provides a more comprehensive understanding of end‐of‐life care competency by integrating statistical results with in‐depth insights from nurses’ clinical experiences, thereby strengthening the interpretability of the findings.

Against this backdrop, this mixed‐methods study aims to identify the factors associated with end‐of‐life care competency among nurses working in departments with high in‐hospital mortality and to gain a deeper understanding of factors associated with end‐of‐life care competency through the integration of quantitative and qualitative findings. End‐of‐life care competency is not only an individual‐level clinical capability but also a key outcome influenced by nursing management and organizational context. Nurse leaders play an important role in shaping the clinical environment through staffing decisions, education and training programs, ethical climate formation, and support systems for nurses working in high‐mortality settings. Therefore, identifying factors associated with nurses’ end‐of‐life care competency has important implications for nursing management practices aimed at improving care quality and supporting workforce development.

## 2. Methods

### 2.1. Research Design and Setting

This study employed an explanatory sequential mixed‐methods design, beginning with a quantitative study aimed at investigating and analyzing the factors influencing end‐of‐life care competency among nurses in departments with high in‐hospital mortality. These departments included ICUs, emergency departments, oncology units, cardiology care units, and respiratory care units. Emergency departments were included based on previous empirical evidence identifying them as clinical settings in which nurses frequently encounter end‐of‐life care situations and patient deaths [3]. This was followed by a qualitative study intended to provide a more detailed explanation of the quantitative findings [30].

According to Creswell [29], the explanatory sequential design involves collecting and analyzing quantitative data in the first phase, followed by a qualitative phase to explain and interpret the quantitative results. This approach is particularly useful when statistical results require further exploration to understand how and why the findings occur. In accordance with the explanatory sequential design, findings from the quantitative phase informed the development of the semistructured interview guide and the focus of the qualitative inquiry.

Qualitative data were collected through focus group interviews to explore participants’ experiences and contextual factors that could not be fully captured through survey data, thereby facilitating a more comprehensive interpretation of the findings. Integration was further achieved at the interpretation level through the comparison and synthesis of quantitative and qualitative findings, providing a more comprehensive understanding of end‐of‐life care competency among nurses working in high‐mortality departments.

### 2.2. Participants and Data Collection

This study included nurses with at least one year of practice in departments with high in‐hospital mortality (ICUs, ERs, oncology units, cardiology care units, and respiratory care units) in one tertiary hospital, one 600‐bed general hospital, and one 200‐bed general hospital located in Gyeongsangnam‐do, Korea. The recruited nurses were directly involved in patient care, understood the study’s purpose, and voluntarily consented to participate.

The G∗Power software (Ver. 3.1.9.7) was used to determine the sample size. In the initial study design, 19 variables were considered. Based on a significance level of 0.05, an effect size of 0.15, a power of 0.90, and 19 predictor variables, the minimum required sample size was 183. Given a 10% dropout rate, 210 questionnaires were distributed, of which 204 were included in the final analysis after excluding six questionnaires with invalid responses. After data collection, only 12 variables were included in the final hierarchical regression analysis based on theoretical relevance.

#### 2.2.1. Quantitative Data

Prior to using the research instruments, we contacted the developers of each instrument via email to obtain permission for their use. The survey was administered between June 13 and July 11, 2024. The final instrument comprised 138 items, including 14 items on general and work‐related characteristics, 28 on end‐of‐life care competency, 34 on shared decision‐making, 20 on ethical competency, 18 on self‐leadership, and 24 on positive psychological capital.

##### 2.2.1.1. End‐of‐Life Care Competency

The End‐of‐Life Care in the ICU (EOL‐ICU) scale, developed by Montagnini, Smith, and Balisterieri [31], is designed to assess the competencies of ICU providers in delivering end‐of‐life care. In this study, the version of the scale translated by Lee [28] was used to evaluate nurses’ competency in end‐of‐life care. The instrument consists of 28 items, each rated on a five‐point Likert scale. Higher scores reflect greater competency in providing end‐of‐life care. The scale demonstrated high internal consistency, with a Cronbach’s alpha of 0.92 in the original study and 0.89 in the present study.

##### 2.2.1.2. Perceptions of Shared Decision‐Making

To assess perceptions of shared decision‐making, this study employed the Shared Medical Decision‐Making Scale for End‐of‐Life Patients in Korea, developed by Jo [32] for nurses caring for patients at the end of life. The scale was modified in this study to incorporate consistent terminology and official naming conventions. It comprises 34 items, each rated on a five‐point Likert scale. Higher scores reflect stronger perceptions of shared decision‐making. The scale demonstrated good internal consistency, with a Cronbach’s alpha of 0.80 in the original study and 0.95 in the present study.

##### 2.2.1.3. Ethical Nursing Competency

The Ethical Nursing Competence Self‐Rating Scale for Clinical Nurses (ENC‐S‐CN), developed by Kang and Oh [33], was used to measure ethical competence in nursing practice. The instrument comprises 20 items, each rated on a four‐point Likert scale. A higher score indicates greater ethical competence. The Cronbach’s alpha was 0.89 in the original study and 0.90 in this study.

##### 2.2.1.4. Self‐Leadership

The instrument used to measure self‐leadership in this study was based on the original Self‐Leadership Questionnaire (SLQ) developed by Manz [34] for leaders and managers within organizations. The SLQ was subsequently revised and adapted for teachers by Kim [35], and later further modified for nurses by Cho [36]. The final version consists of 18 items, each rated on a five‐point Likert scale, with higher scores indicating stronger self‐leadership. The Cronbach’s alpha was 0.89 in Kim’s study [35], 0.86 in Cho’s study [36], and 0.86 in the present study.

##### 2.2.1.5. Positive Psychological Capital

The Psychological Capital Questionnaire (PCQ) was developed by Luthans et al. [37] to measure positive psychological capital in individuals within organizational settings. In this study, the Korean version of the PCQ was used after obtaining permission from Mind Garden, the copyright holder. The instrument consists of 24 items, each rated on a six‐point Likert scale, with higher scores reflecting greater levels of positive psychological capital. The Cronbach’s alpha was 0.89 in the original study by Luthans et al. [37] and 0.94 in the present study.

#### 2.2.2. Qualitative Data

Among the 204 nurses who participated in the quantitative survey, participants for the qualitative phase were identified based on predefined inclusion and exclusion criteria and prior consent to participate in the interview phase. From this pool, 31 nurses initially expressed willingness to participate after being informed about the qualitative interview procedures. These nurses were then contacted individually to confirm their availability and willingness to participate. A two‐stage sampling strategy was applied. First, convenience sampling was used to recruit participants who were readily accessible and willing to participate. Second, purposive sampling was employed to ensure variation in clinical departments and end‐of‐life care experiences in line with the study′s purpose. Ultimately, nine nurses were included in the qualitative study. No participants explicitly refused participation; however, some were unable to participate due to scheduling conflicts prior to the interview arrangement. Participants were assigned to three focus groups based on departmental affiliation (ward, ICU, and ER) to reflect differences in end‐of‐life care experiences and roles across clinical departments.

The inclusion criteria were as follows: (1) nurses with at least 1 year of experience in high‐mortality departments (ICUs, ERs, oncology units, cardiology units, and respiratory units) and experience in providing end‐of‐life care, (2) nurses who understood the purpose of the study and voluntarily agreed to participate in qualitative interviews, and (3) nurses who were able to sufficiently articulate their experiences.

The exclusion criteria were as follows: (1) nurses not directly involved in patient care or nurse managers, (2) those who withdrew during the study, and (3) those with communication difficulties that made in‐depth interviews difficult.

Focus group interviews were selected because end‐of‐life care experiences are closely linked to team‐based interactions in clinical settings [8]. This method enables the generation of rich data through participant interaction and allows for the exploration of both shared and distinct experiences across different clinical departments. To address potential limitations of focus group interviews, such as the dominance of certain participants or limited opportunities for individual expression, the researcher actively facilitated balanced participation and moderated the discussion to prevent any single viewpoint from dominating. In addition, for sensitive topics, follow‐up questions were used to encourage participants to express their personal experiences in greater depth.

Focus group interviews were conducted according to participants’ availability between August 21 and October 10, 2024. Interviews were conducted by a researcher with nursing experience in end‐of‐life care, following established focus group interview guidelines. Each session lasted approximately 60 min, was audio‐recorded with participants’ consent, and was subsequently transcribed. The transcripts were shared with participants to verify the accuracy of the data.

The interview began with self‐introductions to create a positive and open atmosphere. A semistructured questionnaire containing predefined interview questions was used to guide the discussion. Questions were asked in response to participants’ answers, and additional questions were asked if needed.

The main questions were as follows: “Can you describe a memorable episode when caring for patients at the end of life?” “Can you describe a challenging situation you encountered while caring for patients at the end of life?” “Can you share your feelings, thoughts, and lessons learned while caring for patients at the end of life?” “What do you think of the roles of shared decision‐making, ethical competence, and leadership in terms of caring for patients at the end of life?” “Can you share your opinion about additional requirements for enhancing end‐of‐life nursing care, if any?”

The size of each focus group was guided by established methodological literature. Previous studies recommend small groups of fewer than six participants to facilitate in‐depth discussion and active engagement [38]. Mini focus groups of 4–6 participants, or even smaller groups of 3–5 participants, are considered appropriate when exploring sensitive and emotionally intensive experiences [39, 40].

Guest et al. [41] reported that approximately 90% of themes can be identified within three to four focus groups, supporting the feasibility of achieving data saturation with a limited number of groups. In this study, three focus groups were conducted according to departmental characteristics, with each group comprising fewer than six participants. Data collection and analysis were conducted concurrently, and recruitment continued until no new themes or meaningful information emerged. In the later stages, previously identified categories were repeatedly confirmed, and no additional codes or themes were generated. Therefore, data saturation was achieved. Based on these findings and methodological recommendations, the final sample of nine participants was considered sufficient.

### 2.3. Data Analysis

#### 2.3.1. Quantitative Data Analysis

Participants’ general and work‐related characteristics, end‐of‐life care competency, perceptions of shared decision‐making, ethical nursing competency, self‐leadership, and positive psychological capital were summarized using frequencies, percentages, means, and standard deviations. Hierarchical regression analysis was performed to determine factors associated with end‐of‐life care competency. The collected data were analyzed using the IBM SPSS Statistics for Windows, Version 27.0.

#### 2.3.2. Qualitative Data Analysis

Thematic analysis, a qualitative research method developed by Braun and Clarke [42], was used to analyze the qualitative data obtained from the focus group interviews. This method involves a six‐step process: familiarization, coding, generating themes, reviewing themes, defining and naming themes, and reporting findings. Initially, the authors transcribed audio recordings from the focus group interviews to familiarize themselves with the data. Data were organized by transcribing audio recordings immediately after each interview. Repeated words and critical expressions were highlighted throughout the interview. Subsequently, data pertaining to study questions were identified and categorized to generate initial codes. In the third phase, the initial codes and relevant contrasting data were reviewed to generate significant subthemes. In the fourth phase, subthemes were reviewed for the entire dataset, including the coded data. In this phase, previous subthemes were combined, and additional themes were created. In the fifth phase, each theme was analyzed in depth, and core theme names were determined by identifying their scope and boundaries. In the sixth phase, integrated findings comprising key codes, subthemes, and main themes were synthesized.

### 2.4. Ethical Consideration

Participants were provided with a written explanation outlining the study’s objectives, methods, their rights, confidentiality measures, and procedures for data disposal. Written informed consent was obtained prior to participation. To ensure confidentiality, all personal information in the collected data was replaced with unique identifiers and code numbers. The data were used solely for research purposes, retained for 3 years following the completion of the study, and subsequently destroyed. Small tokens of appreciation were given to participants as a gesture of gratitude for their involvement.

### 2.5. Rigor

To ensure the qualitative study’s validity, the criteria identified by Guba and Lincoln [43] were employed. Among the initial participants, those with extensive experience in end‐of‐life care were selected to enhance the credibility of the findings. During the interviews, participants were asked the same question in different ways, and interviews were transcribed by the researcher immediately after each session. The researcher and participants confirmed the transcripts to prevent data loss or distortion. Moreover, transcripts were randomly compared with audio recordings to verify their accuracy. Transferability was ensured by collecting and analyzing data until theoretical saturation was achieved or no further data could be obtained from the participants. When the answers to additional questions and interview statements were ambiguous, the participants were contacted again for questions via phone calls to ensure the clarity of the answers. To maintain consistency, an experienced qualitative researcher participated in the evaluation of the study findings. Lastly, efforts were made to minimize researcher bias by bracketing the researcher’s preconceptions during data collection and analysis.

## 3. Results

### 3.1. Quantitative Findings

#### 3.1.1. Participants’ General and Work‐Related Characteristics

Most participants were female and unmarried, accounting for 91.2% (*N* = 186) and 62.3% (*N* = 127), respectively, and the mean age was 31.4 years. Most participants (*N* = 160, 78.4%) held a bachelor’s degree, followed by an associate’s degree (*N* = 24, 11.8%) and a master’s degree (*N* = 20, 9.8%). The average length of participants’ clinical experience was 7.75 years in total and 5.13 years in the current hospital. Most participants (*N* = 67, 32.8%) were working in respiratory/cardiology care units, followed by ICUs (*N* = 58, 28.4%), oncology units (*N* = 48, 23.5%), and ERs (*N* = 31, 15.2%). The mean number of deaths experienced by participants in the last 3 months was 3.99 ± 5.25. Most participants did not have training in hospice care, accounting for 64.2% (*N* = 131), and 73 (35.8%) had training in hospice care. Most participants (*N* = 26, 35.6%) had taken hospice‐related courses during their undergraduate or graduate studies, followed by continuing education (*N* = 18, 24.7%), at least two courses at different institutions (*N* = 11, 15.1%), work‐related training (*N* = 9, 12.3%), and training provided from hospice (*N* = 6, 8.2%). A majority of participants (*N* = 157, 77.0%) experienced the death of family members or friends (Table 1).

**TABLE 1:** Participants’ general and work‐related characteristics (*N* = 204).

Characteristics	Classification	*n*	%	Mean ± SD	Range
Age (years)	≤ 25	42	20.6	31.41 ± 6.74	22∼53
	26∼30	71	34.8		
	31∼35	40	19.6		
	≥ 36	51	25.0		

Sex	Female	186	91.2		
	Male	18	8.8		

Marital status	Single	127	62.3		
	Married	77	37.7		

Education level	Associate’s degree	24	11.8		
	Bachelor’s degree	160	78.4		
	Master’s degree or higher	20	9.8		

Religion	None	63	30.9		
	Yes	141	69.1		

Total clinical experience	1 to < 3 years	51	25.0	7.75 ± 5.70	1.00∼26.83
	3 to < 5 years	30	14.7		
	5 to < 10 years	58	28.4		
	10 years or more	65	31.9		

Years of work experience at the current job	1 to < 3 years	81	39.7	5.13 ± 4.31	1.00∼21.00
	3 to < 5 years	37	18.1		
	5 to < 10 years	63	30.9		
	10 years or more	23	11.3		

Work department	Respiratory/cardiology care unit	67	32.8		
	Oncology unit	48	23.5		
	Emergency room	31	15.2		
	ICU	58	28.4		

Number of deaths experienced in the last 3 months	< 1	30	14.7	3.99 ± 5.25	0∼40
	1∼4	107	52.5		
	5∼9	43	21.1		
	≥ 10	24	11.8		

Previous hospice care training	Yes	73	35.8		
	No	131	64.2		

Educational method (hospice/palliative care education)	Courses taken during undergraduate/graduate studies	26	35.6		
	Job training	9	12.3		
	Continuing training	18	24.7		
	Hospice facility	6	8.2		
	Internet	1	1.4		
	Academic conference	2	2.7		
	Duplicate	11	15.1		

Experience of the death of family members or friends	No	47	23.0		
	Yes	157	77.0		

Total		204	100		

#### 3.1.2. Participants’ Level of Key Variables

The mean score of perception of shared decision‐making was 4.38 ± 0.38 out of 5, and the mean score of ethical nursing competency was 3.17 ± 0.33 out of 4. The mean score of self‐leadership was 3.44 ± 0.50 out of 5, and the mean score of positive psychological capital was 4.17 ± 0.69 out of 6. The mean score of end‐of‐life care competency was 3.62 ± 0.44 out of 5.

#### 3.1.3. Factors Associated With End‐of‐Life Care Competency

Hierarchical regression was performed using variables with significant correlations to identify factors associated with end‐of‐life care competency among nurses. As tolerance was greater than 0.1 and the variance inflation factor was lower than 10, multicollinearity did not exist. Moreover, the Durbin–Watson statistic was 1.913, which was close to 2, suggesting that there was no autocorrelation in the residuals. Perception of shared decision‐making (*β* = 0.20, *p* = 0.002), ethical nursing competency (*β* = 0.31, *p* < 0.001), and positive psychological capital (*β* = 0.19, *p* = 0.030) were shown to be factors associated with end‐of‐life care competency. The explanatory power was 46.8% (*F* = 14.04, *p* < 0.001, *R* ^2^ = 0.468, Adj‐*R* ^2^ = 0.435) (Table 2).

**TABLE 2:** Effect of perception of shared decision‐making, ethical nursing competency, self‐leadership, and positive psychological capital on end‐of‐life care competency (*N* = 204).

	Model 1					Model 2				
	*B*	SE	*β*	*t*	*p*	*B*	SE	*β*	*t*	*p*
Constant value	3.33	0.12		28.81	< 0.001	0.27	0.31		0.88	0.378
Marital status (based on single)										
Married	0.05	0.07	0.06	0.72	0.470	0.01	0.05	0.01	0.14	0.890
Work experience at the current hospital (based on 1 to < 3 years)										
3 to < 5 years	0.03	0.09	0.02	0.30	0.764	−0.02	0.07	−0.01	−0.24	0.811
5 to < 10 years	−0.01	0.08	−0.01	−0.13	0.895	−0.06	0.06	−0.06	−0.95	0.341
10 years or longer	0.21	0.11	0.15	1.81	0.072	0.05	0.09	0.04	0.56	0.576
Number of deaths experienced (based on none)										
1–4	0.16	0.09	0.18	1.74	0.084	0.13	0.07	0.15	1.85	0.066
5–9	0.25	0.11	0.23	2.31	0.022	0.19	0.08	0.18	2.31	0.022
10 or more	0.18	0.12	0.13	1.45	0.148	0.09	0.10	0.07	0.94	0.350
Hospice education (based on no)										
Yes	0.10	0.07	0.10	1.46	0.147	0.03	0.05	0.04	0.66	0.510
Perception of shared decision‐making						0.24	0.07	0.20	3.21	0.002
Ethical nursing competency						0.41	0.09	0.31	4.46	< 0.001
Self‐leadership						0.10	0.08	0.12	1.35	0.178
Positive psychological capital						0.12	0.06	0.19	2.19	0.030
Adj‐*R* ^2^	0.053					0.435				
*R* ^2^	0.090					0.468				
*R* ^2^ change	—					0.378				
*F* (*p*)	2.43 (0.016)					14.03 (< 0.001)				
*F* change (*p*)	—					33.95 (< 0.001)				

*Note:* Durbin–Watson = 1.913; tolerance = .352∼.894; VIF = 1.118∼2.842.

### 3.2. Qualitative Findings

In the qualitative study, nine participants were recruited, comprising three nurses from each of the following units: medical, ICU, and ER. All participants were female, with ages ranging from 27 to 36 years (*M* = 30.11 years). Their total clinical experience varied from 2 years and 1 month to 13 years and 4 months (*M* = 6.96 years), and experience within their current units ranged from 2 years and 1 month to 6 years (*M* = 3.65 years). On average, participants reported 9.11 patient deaths over the past 3 months (Table 3).

**TABLE 3:** Characteristics of participants in the qualitative study (*N* = 9).

No	Group	Sex	Age (years)	Total clinical experience (years, months)	Current department experience (years, months)	End‐of‐life care frequency (over the past 3 months, times)
1	ICU	F	27	5 years 5 months	3 years 2 months	6
2	ICU	F	29	7 years 3 months	3 years 7 months	6
3	ICU	F	35	13 years 4 months	2 years 9 months	5
4	Ward	F	27	2 years 1 month	2 years 1 month	10
5	Ward	F	29	4 years 6 months	4 years 6 months	12
6	Ward	F	30	6 years 7 months	2 years 3 months	6
7	ER	F	28	4 years 6 months	4 years 6 months	15
8	ER	F	30	6 years	6 years	12
9	ER	F	36	13 years	4 years	10

The qualitative findings were organized to capture the contextual processes through which nurses construct and sustain end‐of‐life care competency in clinical practice. Five main themes and 12 subthemes were derived (Table 4). These findings elaborate on the processes, meanings, and contextual conditions underlying each area of end‐of‐life care competency identified in the quantitative phase, which could not be captured through statistical relationships alone.

**TABLE 4:** Themes and subthemes from qualitative content analysis.

Theme	Sub‐theme	Contents
Efforts made to improve the quality of end‐of‐life care	Applying knowledge gained from professional and clinical experiences	Early detection and prompt intervention for end‐of‐life symptoms
		Performing basic skilled nursing care
	Creating an environment for caring for patients at the end of life	Providing a private room for end‐of‐life farewells between patients and their loved ones
		Creating familiar environments for patients
		Providing care in a setting that allows for greater comfort and movement
	Wishes for better end‐of‐life care	Requiring education on end‐of‐life care
		Requesting more workers in departments with high mortality
		Expanding support for overcoming burnout at organizational levels

Involving everyone in the decision‐making process as a key factor	Utilizing a support system for sharing information	Acquiring treatment direction through counseling
		Sharing and verifying the decisions on life‐sustaining treatment through various routes
	Guiding patients to actively participate in decision‐making	Providing sufficient explanation for self‐determination and aiding in making their own decisions
		Promoting patient stability during participation
		Acceptable attitudes toward a patient’s decision
	Encouraging family involvement in care	Sharing information between family members and encouraging them to be involved in decision‐making
		Promoting an opportunity for decision‐making through patient visit

A moral compass found in the mists	Moral distress due to ethical value conflicts	Doubting if the use of restraints is necessary
		Different opinions about palliative sedation
		Contemplating the exclusion of patient’s opinion when withdrawing life‐sustaining treatment
	Growth through ethical reflection	Self‐reflection on feeling relief upon receiving an order to withdraw life‐sustaining treatment
		Taking action in accordance with ethical standards when problems arise

A solitary journey toward self‐directed leadership	Acquiring and exhibiting leadership	Monitoring and emulating role models
		Mentoring junior colleagues for their professional growth
	Independent thinking and practical action	Making an effort to prevent mistakes
		Organizing thoughts to guide behavior and maintain mental clarity
		Providing nursing care with the thought that there is only “me”

Power of positive thinking that protects the inner light	Efforts to maintain stability and balance	Participating in work with positive attitudes
		Accepting unpredictable situations in the clinical setting
		Separating work life from personal life
	Utilizing strategies for resilience	Sharing similar experiences with colleagues
		Engaging in various forms of self‐rewarding activities

#### 3.2.1. Main Theme 1: Efforts Made to Improve the Quality of End‐of‐Life Care

This theme describes how end‐of‐life care competency is enacted through accumulated clinical experience and continuous adaptation to clinical situations, reflecting a process in which nurses actively adjust their practice to provide appropriate care within environmental and organizational constraints.

##### 3.2.1.1. Subcategory 1: Applying Knowledge Gained From Professional and Clinical Experiences

The participants demonstrated their ability to rapidly detect and respond to symptoms in patients at the end of life based on their clinical experiences and made an effort to preserve patient dignity.

*“There are clinical signs of impending death… (ellipsis). In that case, I take action following the patient’s treatment direction.” (Participant 2)*

*“I think the quality* of *care can be improved by paying attention to patients at the end of life and assisting them with fundamental nursing care*. *Even just meeting the physiological needs, which are one of the five basic human needs, makes patients feel very satisfied.” (Participant 3)*

##### 3.2.1.2. Subcategory 2: Creating an Environment for Caring for Patients at the End of Life

Participants described adapting the care environment to support patients and families during the end‐of‐life process, particularly within multipatient room settings where privacy is limited. Efforts were made to create more comfortable and meaningful spaces within existing structural constraints.

*“I prepared a separate space for patients and their families to have their own private moment. In the room, they talked to each other saying ‘Thank you for raising me. I hope we meet in the next life too’… I think the bereaved can stand up to the loss when they were given sufficient time.” (Participant 5)*

*“I thought that creating a familiar environment would help patients become emotionally stable. So, I called a chaplain or played religious music when patients wanted to pray.” (Participant 3)*

*“It’s not easy for me to use equipment in multi-patient rooms because the room is so small. But only one patient stays in an end-of-life care room (ellipsis) it’s easy for me to care for the patient.” (Participant 5)*

##### 3.2.1.3. Subcategory 3: Wishes for Better End‐of‐Life Care

Participants expressed the need for further support in end‐of‐life care practice, including opportunities for professional education, communication skill development, and organizational resources to manage workload and emotional burden.

*“I thought that I need to know more so that I can explain things well to patients.” (Participant 8)*

*“For employees, the hospital provided training regarding communication skills required for each stage of life according to their years of clinical experience. (ellipsis) Although training in communicating between professions, interviewing patients or caregivers, and breaking bad news is required for all employees, training should be especially provided to those with less years of experience.” (Participant 9)*

*“It’s not easy for me to care for patients at the end of life while caring for other patients. I hope there’s an end-of-life support team, a team providing end-of-life counseling, or there are more workers in the department.” (Participant 5)*

*“I hope there are more active burnout prevention programs for nurses who frequently experience patient deaths. A few years ago, the hospital provided healthcare staff who were experiencing high levels of burnout while caring for COVID-19 patients with time to watch a movie. While we were watching the movie, we could talk to each other to resolve emotional baggage. We could feel that the hospital care about us and were consoled.” (Participant 6)*

#### 3.2.2. Main Theme 2: Involving Everyone in the Decision‐Making Process as a Key Factor

This theme reflects how decision‐making in end‐of‐life care is experienced as a collaborative and structured process involving communication, information sharing, and family engagement in clinical practice.

##### 3.2.2.1. Subcategory 1: Utilizing a Support System for Sharing Information

Participants reported that end‐of‐life counseling clarifies the direction of nursing care. Patients’ decisions on life‐sustaining treatment are shared and continuously verified through various routes, including electronic medical records (EMRs), a computerized information system, multidisciplinary discussions, and a communication process during shift handover.

*“We offered a counseling service if patients wanted to have counseling for more sensitive topics, such as life-sustaining treatment or a referral to hospice. During the counseling session, the patients made future directions in the treatment.” (Participant 5)*

*“In the EMRs, patients’ decisions for future directions in the treatment are shared in the EMRs expressing as R (Do Not Resuscitate; DNR), a (physician order for life-sustaining treatment), and p (advance directives). We shared and reverified each other’s ideas at meetings…. (ellipsis). We checked the main decision maker and completed shift handover.” (Participant 6)*

##### 3.2.2.2. Subcategory 2: Guiding Patients to Actively Participate in Decision‐Making

Participants reported that sufficient explanation is required for decision‐making, which relieves anxiety and promotes stability. In addition, with sufficient explanation and time, patients and their families were more likely to accept end‐of‐life care.

*“First, I asked them whether they want to use mechanical ventilators, be transferred to the ICUs, or be on life-sustaining treatment. (ellipsis) I gave them sufficient time to think.” (Participant 8)*

*“Those who were exposed to treatment decision-making were more likely to understand and accept their end of life, which reduced psychological distress. They know whereof their own bad conditions…, they felt a sense of stability as they made decisions by themselves.” (Participant 5)*

##### 3.2.2.3. Subcategory 3: Encouraging Family Involvement in Care

Participants recognized the importance of family involvement in providing patient‐centered nursing care, particularly within the cultural context of Korea. Accordingly, they encouraged family visits to facilitate meaningful discussions and support the decision‐making process. During visiting hours, family members were given the opportunity to participate in care decisions and were encouraged to share information among themselves.

*“Most caregivers do not know patients’ conditions… (ellipsis) when I explain to a patient, I suggest them to share information between family members and discuss their opinions, even although the patient’s decision is important.” (Participant 8)*

*“Despite the limited visiting hours in the ICU…, we allow a family to visit a dying patient. (ellipsis) If the family cannot visit the patient, we often help the patient to make conversation on the phone.” (Participant 1)*

#### 3.2.3. Main Theme 3: A Moral Compass Found in the Mists

This theme describes nurses’ lived experiences of ethical tension and reflection when providing end‐of‐life care in situations involving conflicting values and clinical constraints.

##### 3.2.3.1. Subcategory 1: Moral Distress due to Ethical Value Conflicts

Although participants provided life‐sustaining care by disregarding the patient’s opinion and used restraints and palliative sedation following the physician’s orders, they were deeply concerned about the patient’s loss of dignity during the process. They reported that these concerns broadened their perspectives.

*“Most patients experience delirium in their last days, and those with severe delirium are very aggressive and cannot control their behaviors. (ellipsis) Patients are usually forced to be physically restrained while they are conscious… which is always a dilemma for me although I get consent. I felt that I don’t treat the patient as a person. That’s not to say I will not use restraints because I cannot secure patient’s safety without restraints.” (Participant 5)*

*“Patients under sedation seemed to be comfortable… but I was worried that I might speed up the death. Sometimes, caregivers ask whether sedation accelerates the death…. One day, I found out that the patient I had given medication to had died by the time I started work the next morning. So, it’s not easy for me to initiate sedation therapy. Now, I know that it’s not true, but those who had the medication not working for them sometimes take different medications or high-dose medications…. In that situation, I always wondered if this was the right?” (Participant 4)*

*“We continue giving medications to unconscious patients with no family or friends because we cannot get consent for a DNR order…. I kept wondering if this is the right intervention…. I kept asking myself if the person is going to survive or die? if I’m doing the right thing?” (Participant 7)*

##### 3.2.3.2. Subcategory 2: Growth Through Ethical Reflection

When the decision to withdraw life‐sustaining treatment was made, participants felt relieved that they would be free from work‐related burdens, such as emergency situations. Simultaneously, they reflected on their sense of relief and regretted experiencing such feelings. They also contemplated the meaning of a dignified death. Moreover, they made an effort to preserve the autonomy and dignity of patients in a conflicting situation in accordance with ethical standards.

*“I felt relieved when I received a DNR order, reducing burdens. The patient was no longer my priority to care…. I was disappointed at my thought. I reflect on it myself whenever it happens. And, I also think ‘what more can I do for the patient?’ (ellipsis) because it′s a very last important and precious moment for the patient.” (Participant 9)*

*“The decision to withdraw life-sustaining treatment does not mean that I am giving up on patients. I take action, if necessary… (ellipsis) if patients refuse to receive a blood transfusion due to their religious belief, it’s impossible to convince them. As an alternative approach, we could initiate IV hydration therapy or iron supplements.” (Participant 5)*

#### 3.2.4. Main Theme 4: A Solitary Journey Toward Self‐Directed Leadership

This theme illustrates how leadership emerges and is developed through role modeling, mentoring, and independent clinical practice among nurses in end‐of‐life care settings, reflecting how leadership is constructed in everyday clinical interactions rather than functioning as a static individual trait.

##### 3.2.4.1. Subcategory 1: Acquiring and Exhibiting Leadership

Participants observed expert nurses in clinical practice as role models and made efforts to emulate their behaviors and attitudes. To become role models for junior colleagues, they provided support and mentorship.

*“I met my role model when I encountered a caregiver with no emotional control. The role model teaches me know-how of communication and helps me how to overcome work pressure. She told me to let her know anything that I cannot handle…. I thought that I want to be her. So, whenever I work with junior colleagues, I try to act just like her.” (Participant 4)*

*“I always open my eyes and ears. So, I can help my junior colleagues whenever things happen to them… Because patients at the end of life and their caregivers are exhausted and sensitive, I praised junior colleagues when they handle a situation well. And, I help and give them feedback when I think, ‘why did they say that?’” (Participant 9)*

##### 3.2.4.2. Subcategory 2: Independent Thinking and Practical Action

Participants made conscious efforts to avoid repeating mistakes encountered during end‐of‐life care by employing their own strategies. They engaged in self‐reflection, organized their thoughts, and regulated their emotions with a sense of personal initiative. Moreover, they approached patients with responsibility and autonomy, believing that they were primarily responsible for providing care.

*“I kept recording and thinking about mistakes that I’ve made. For example, of course, I see many patients at the end of life, so it feels routine to me, but for them, it’s not. But, I didn’t have any empathy… I shouldn’t do that because I didn’t have time to do so…I feel worthy and healed when I don’t repeat the same mistakes.” (Participant 7)*

*“I make up my mind to do better next time and to care more and encourage myself. I hypnotize myself to think that ‘only I can care for all of these patients.’ With that thought, I can go to the patients more often even if I am tired, (ellipsis) if I behave with responsibility, I can anticipate better by thinking what are the things I need to improve? I need such supplementary actions next time.’” (Participant 8)*

#### 3.2.5. Main Theme 5: Power of Positive Thinking That Protects the Inner Light

This theme reflects how nurses sustain emotional stability and resilience through adaptive coping strategies and cognitive reframing in end‐of‐life care situations.

##### 3.2.5.1. Subcategory 1: Efforts to Maintain Stability and Balance

Participants approached their work with a positive mindset and accepted various unpredictable situations in clinical settings. With that attitude, they actively sought stability and explored various strategies to maintain a healthy balance between their personal and professional lives.

*“When I first cared for a patient at the end of life, I kept thinking about ‘what have I done wrong?’ or ‘will it be okay?’ even though I was at home. After getting off from work, I asked the next shift nurse about the patient… Now, I know a patient’s condition can change frequently and accept it as normal.” (Participant 5)*

*“Recently, I mostly forget about the patients once I change clothes and get out of the hospital. I can’t change the past. I try not to keep it in mind. So, I’m able to return to work again.” (Participant 9)*

##### 3.2.5.2. Subcategory 2: Utilizing Strategies for Resilience

Participants engaged in self‐care and adopted coping strategies to care for themselves and cope with the challenges of caring for end‐of‐life patients by sharing experiences with colleagues or providing themselves with material, physical, and psychological rewards.

*“My colleagues show empathy because they’ve been already there and understand me well, which helps me recover.” (Participant 4)*

*“Even if I’m tired, taking a short break to have meals or snacks makes me feel refreshed. By doing so, I’m less stressed for the rest of the day. Moreover, retail therapy also plays a role. (ellipsis) I feel much better if I take a rest. After taking a good rest, I hardly remember my feelings and events that previously bothered me.” (Participant 5)*

### 3.3. Integration of Quantitative and Qualitative Findings

The integration of quantitative and qualitative findings demonstrated both convergence and divergence in factors associated with end‐of‐life care competency among nurses in high‐mortality departments.

Shared decision‐making, ethical nursing competency, and positive psychological capital, which were identified as significant predictors in the quantitative phase, were also reflected in the qualitative findings in a consistent manner. Shared decision‐making was described in relation to information sharing, multidisciplinary communication, and family involvement in decision‐making processes. Ethical nursing competency was reflected in accounts of moral distress and ethical reflection in clinical situations. Positive psychological capital was reflected in descriptions of emotional regulation, cognitive coping, and peer support.

In contrast, self‐leadership showed divergence between the two strands. Although it was not statistically significant in the quantitative phase, the qualitative findings described self‐leadership in terms of reflective practice, independent clinical actions, and role modeling within clinical settings.

Overall, the qualitative findings illustrated how each variable is practically experienced and diversely manifested in clinical settings, thereby serving to complement and contextualize the quantitative results.

## 4. Discussion

This study employed an explanatory sequential mixed‐methods design to identify and analyze factors associated with end‐of‐life care competency among nurses in departments with a high incidence of end‐of‐life cases. Nurses in departments with a high incidence of end‐of‐life cases accumulate extensive experience in providing end‐of‐life care. These experiences have been shown to reduce their fear of caring for dying patients and enhance their overall competence in end‐of‐life care [4, 8, 44]. Qualitative findings were used to describe nurses’ clinical experiences and practice contexts in high‐mortality settings. As shown in the qualitative analysis, the findings are supported by nurses’ efforts to provide skilled nursing care based on clinical experiences and to establish environments that ensure a dignified death. However, the challenges encountered during end‐of‐life care can cause significant psychological stress for nurses, necessitating the development of various coping strategies [8]. One such strategy identified in the qualitative analysis, “Wishes for better end‐of‐life care,” reflects nurses’ motivation to sustain their professional performance. This is particularly relevant for newly graduated nurses, who often face heavy workloads and heightened stress due to limited experience in end‐of‐life care [45]. Therefore, systematic education and support should be provided before they are assigned to end‐of‐life patients. A multidimensional approach to improving end‐of‐life care competencies, such as hospice and palliative care training, simulation‐based education, and mentoring programs, is essential. In comparison with previous studies, shared decision‐making has been consistently identified as a key element in improving end‐of‐life care outcomes [46–48]. The qualitative findings illustrate how related clinical practices are experienced in context.

Nurses play a crucial role in facilitating communication between patients and healthcare providers during end‐of‐life treatment decision‐making and participate in shared decision‐making to improve patients’ quality of life [46–48]. In the qualitative analysis of high‐mortality departments, nurses shared information through multidisciplinary discussion, sought treatment direction, encouraged patients and their families to be involved in patient‐centered decision‐making, and demonstrated attentive listening and reflection. Effective communication skills, grounded in ethical awareness, are essential for performing these roles [7, 11]. In particular, communication skills in end‐of‐life care, including the delivery of sensitive information such as prognosis and treatment options, are important professional competencies in supporting patients and their families [49]. Therefore, these communication competencies may be considered in undergraduate nursing curricula and continuing education programs for nurses. However, the qualitative findings should be interpreted in light of the Korean clinical context, where end‐of‐life decision‐making is often family‐centered and patients may be indirectly involved in the process. In addition, they should be understood as contextual descriptions of how shared decision‐making is operationalized in clinical practice rather than as a comprehensive representation of all theoretical domains of the construct. Accordingly, they are presented as contextual accounts of clinical practice.

Beyond decision‐making processes, ethical challenges were also described as an important aspect of end‐of‐life care. Seo and Yeom [50] reported that ethical dilemmas contribute to psychological burnout among nurses, and that end‐of‐life care competency increases as the level of psychological dilemma decreases. Similarly, Wu et al. [20] revealed that nurses who care for patients at the end of life are more likely to experience a “negative emotional environment,” resulting in psychological distress caused by a stressful work environment and high workload. Accordingly, they emphasized the importance of setting emotional boundaries and leadership. In line with previous studies, the present findings also indicate that ethical nursing competency is closely linked to continuous ethical reflection and moral reasoning in clinical practice. While previous studies have primarily emphasized stress reduction through emotional boundary setting, the present study extends this perspective by suggesting that ethical reflection may also function as a process for strengthening ethical nursing competency.

In the qualitative analysis of this study, nurses created their own emotional boundaries by engaging in ethical reflection and resolving moral dilemmas. They also demonstrated ethical behaviors by considering various alternatives when faced with challenging situations. These findings suggest a key mechanism for enhancing ethical nursing competency, ultimately contributing to improved end‐of‐life care competency. These findings highlight the need not only for individual effort but also for organizational support to facilitate opportunities for ethical reflection, promote the sharing of ethical case discussions, and implement structured ethical education programs to foster a strong ethical culture in clinical settings. These findings highlight the need for organizational support, including ethics consultation opportunities, case discussion, and structured ethics education.

Nurses caring for patients at the end of life experience various levels of work‐related stress, which can negatively impact their quality of care [20, 51]. Positive psychological capital acts as a critical resource for maintaining emotional stability and sustaining nursing competency in such high‐stress environments [4, 13]. Consistent with previous literature, positive psychological capital was confirmed in this study as an important psychological resource supporting resilience and emotional stability among nurses.

In this study, nurses attempted to continue caring for patients at the end of life by using their positive psychological capital as recovery resources. A study by Chen et al. [51] reported that leisure activities provided in the workplace significantly alleviated work stress and promoted sociopsychological well‐being, emphasizing the need to cultivate an organizational culture that enables the expression and development of positive psychological capital. These organizational supports are expected to improve not only end‐of‐life care competency but also the quality of care by strengthening nurses’ expertise. In this context, the qualitative findings are presented as contextual descriptions of coping processes related to positive psychological capital in clinical practice.

This study found no significant effect of self‐leadership on end‐of‐life care competency, which is inconsistent with previous studies reporting self‐leadership as a positive factor in nursing performance and professional development [12]. However, Pursio et al. [12] reported that nurses display self‐leadership by expanding their knowledge, developing skills, and fostering positive attitudes through clinical experiences, which contribute positively to nursing practice and well‐being. Furthermore, nurses’ communication skills and organizational factors, such as the leadership styles of nurse managers, influence the development of self‐leadership. The study emphasizes that self‐leadership is influenced by the organizational environment and structural factors collectively, not solely by personal ability. For instance, a manager’s leadership style, interaction between members, and opportunities for participation in decision‐making are key organizational conditions for developing self‐leadership [12]. Although the quantitative analysis of this study did not identify self‐leadership as a significant factor in end‐of‐life care competency, the qualitative findings indicated that self‐leadership was described by participants in relation to role modeling, mentoring experiences, and independent clinical actions within end‐of‐life care settings. Given that leadership‐related prompts were included in the interview guide, these accounts reflect participants’ reported experiences rather than independently emerging themes. Accordingly, the qualitative findings are presented as contextual descriptions of how leadership‐related experiences were expressed in clinical practice. Based on these descriptive insights, future research should explore whether cultivating an organizational culture of leadership, implementing mentoring systems, or developing practical educational programs could support the autonomous practice of self‐leadership and ultimately enhance end‐of‐life care competency.

This study provides significant practical and integrated insights for enhancing end‐of‐life care competency among nurses. However, several limitations should be noted. The study sample was limited to nurses working in high‐mortality departments of three hospitals located in specific regions of Korea. Consequently, the findings may not be generalizable to all clinical nurses working in diverse geographic areas or hospitals of varying sizes. In addition, the cross‐sectional design of this study limits the ability to infer causal relationships among study variables. Furthermore, as all major variables were measured using self‐reported questionnaires, the possibility of common method variance and social desirability bias should be considered. The inclusion of emergency departments as high‐mortality units may have introduced heterogeneity in end‐of‐life care experiences across clinical settings, which should be considered when interpreting the findings. Although this study employed an explanatory sequential mixed‐methods design, the qualitative findings were derived from a subset of participants within a limited number of institutions, which may limit the transferability of the qualitative results to broader clinical contexts. In addition, the relationships between shared decision‐making, ethical nursing competency, self‐leadership, positive psychological capital, and end‐of‐life care competency among nurses in high‐mortality settings remain insufficiently explored. Therefore, further research is needed to replicate and validate the findings of this study across broader and more diverse populations. Future research should also consider longitudinal designs and multisource or objective assessments to strengthen causal inference and reduce potential measurement bias, thereby improving the robustness of findings.

### 4.1. Implications for Nursing Management and Leadership

This study has several implications for nursing leadership. Nurse managers play a critical role in strengthening end‐of‐life care competency through structured education and training programs that enhance nurses’ shared decision‐making skills, ethical competence, and psychological resources. In addition, fostering a supportive ethical climate and promoting open communication within clinical teams may facilitate better decision‐making processes in end‐of‐life care. Furthermore, in high‐mortality settings such as ICUs and emergency departments, nursing leaders should consider workforce development strategies that support nurses’ emotional resilience and reduce burnout, thereby improving the overall quality of end‐of‐life care delivery.

## 5. Conclusion

This study identified factors associated with end‐of‐life care competency among nurses working in departments with a high incidence of patient deaths and proposed a strategic direction for improving the quality of end‐of‐life care through an in‐depth analysis of clinical experiences. The findings suggest the need for programs that enhance end‐of‐life care competency by focusing on perceptions of shared decision‐making, ethical nursing competency, and positive psychological capital, all of which are key elements in preserving patients’ dignity in death and improving their quality of life. Furthermore, although nurses in high‐mortality departments strive for self‐development of their competencies, personal efforts have limitations. Therefore, organizations should provide practical support, including staffing adjustments, targeted education, and leadership development. In addition, a multilayered strategy is essential to establish effective policy initiatives and structural support systems that reflect nurses’ experiences.

## Author Contributions

So Hyeon Yeon: conceptualization, data collection, formal analysis, and writing–original draft. Kyung Im Kang: data analysis, writing–review and editing and supervision.

## Funding

No funding was received for this study.

## Ethics Statement

This study was reviewed and approved by the Institutional Review Boards of Gyeongsang National University Hospital (GNUH 2024‐05‐022‐001) and Gyeongsang National University (GIRB‐A24‐NY‐0048).

## Conflicts of Interest

The authors declare no conflicts of interest.

## Data Availability

The data used to support the findings of this study are not available because the participants did not provide written consent for their data to be shared publicly.

## References

[1] Kang Ji Y. , Whither Does Caring for the Dying Turn?: Relational-Generative Care at the End of Life, Korean Cultural Anthropology. (2024) 57, no. 1, 3–54.

[2] Statistics Korea , Deaths for 19 Chapters of Causes by Place of Death, 2024, https://kosis.kr/publication/publicationthema.do.

[3] Seo M. J. , Kim J. Y. , Kim S. , and Lee T. W. , Nurses Attitudes Toward Death, Coping With Death and Understanding and Performance Regarding EOL Care: Focus on Nurses at ED, ICU and Oncology Department, Korean Journal of Hospice and Palliative Care. (2013) 16, no. 2, 108–117, 10.14475/kjhpc.2013.16.2.108.

[4] Koo Y. H. and Hae Y. M. , Factors Affecting Performance of Terminal Care for Clinical Nurses: Positive Psychological Capital, Terminal Care Stress and Perception of Patient-Family-Centered Care, Journal of the Korean Data Analysis Society. (2023) 25, no. 5, 1923–1940, 10.37727/jkdas.2023.25.5.1923.

[5] Lee Ju H. and Hwang K. K. , End-of-Life Care for End-Stage Heart Failure Patients, Korean Circulation Journal. (2022) 52, no. 9, 659–679, 10.4070/kcj.2022.0211. 36097835 PMC9470494

[6] National Health Service , End of Life Care, National Health Service. (2022) https://www.nhs.uk/conditions/end-of-life-care/.

[7] Lee E. Y. , Ji H. J. , and Cho J. S. , Roles of Nurses in Decision-Making to Withhold or Withdraw Life-Sustaining Treatment for Patients According to the Life-Sustaining Treatment Decision-Making Act, Bio, Ethics and Policy. (2021) 5, no. 1, 97–114, 10.23183/konibp.2021.5.1.005.

[8] Bautista M. C. M. , Indicar N. A. , Suarez R. F. , and Narvaez R. A. , Nightingale by the Death Bed: A Review on Nurses’ Role and Experiences in Death and Dying, International Journal of Palliative Nursing. (2024) 30, no. 11, 578–590, 10.12968/ijpn.2024.30.11.578. 39621525

[9] Kim H. W. and Kwon S. H. , Effects of Death Anxiety and Perceived end-Of-Life Care Competencies on Fear of Terminal Care Among Clinical Nurses, Journal of Hospice and Palliative Care. (2023) 26, no. 4, 160–170, 10.14475/jhpc.2023.26.4.160. 38075590 PMC10703559

[10] Hökkä M. , Pereira S. M. , Pölkki T. , Kyngäs H. , and Hernández-Marrero P. , Nursing Competencies Across Different Levels of Palliative Care Provision: A Systematic Integrative Review With Thematic Synthesis, Palliative Medicine. (2020) 34, no. 7, 851–870, 10.1177/0269216320918798. 32452294

[11] Hatefimoadab N. , Cheraghi M. A. , Benton D. C. , and Pashaeypoor S. , Ethical Advocacy in the end-Of-Life Nursing Care: A Concept Analysis, Nursing Forum. (2022) 57, no. 1, 127–135, 10.1111/nuf.12656. 34549431

[12] Pursio K. , Kvist T. , Kankkunen P. , and Fennimore L. A. , Self-Leadership and Why it Matters to Nurses: A Scoping Review, International Nursing Review. (2025) 72, no. 1, 10.1111/inr.70014. PMC11881033 40042062

[13] Jung S. Y. and Jeong H. K. , Influence of Positive Psychological Capital and Death Awareness on Terminal Care Performance of Hematooncology Unit Nurses, Journal of Hospice and Palliative Care. (2019) 22, no. 2, 77–86, 10.14475/kjhpc.2019.22.2.77.

[14] Kim L. H. , Kim Su Y. , Kim S. et al., A Mixed-Method Study for Exploring the Difficulties in end-Of-Life Care and end-Of-Life Care Competency in Nurses Who Take Care of Cancer Patients, Asian Oncology Nursing. (2021) 21, no. 2, 98–109, 10.5388/aon.2021.21.2.98.

[15] Son S. Y. and Jeon Mi K. , Factors Influencing end-Of-Life Care Competency of Long Term Care Hospital Nurses: A Cross Sectional Study, Journal of Korean Gerontological Nursing. (2022) 24, no. 2, 174–184, 10.17079/jkgn.2022.24.2.174. PMC12384292 41583099

[16] Jeong D. I. and Eun Y. , Factors Affecting the End-Of Life Care Competency of Tertiary Hospital Nurses, Journal of Hospice and Palliative Care. (2020) 23, no. 3, 139–150, 10.14475/kjhpc.2020.23.3.139. PMC10332723 37497367

[17] Kim D. Y. , Lee H. J. , Yu S.-Y. et al., Awareness of Doctors’ Shared Decision-Making in Life-Sustaining Care Decisions, Journal of Hospice and Palliative Care. (2021) 24, no. 4, 204–213, 10.14475/jhpc.2021.24.4.204. 37674642 PMC10180072

[18] Yoon Y. R. and Bae H. A. , Shared Decision-Making and Informed Consent Legislation in Clinical Decision Making, Korean Journal of Medical Ethics. (2024) 27, no. 2, 71–87, 10.35301/ksme.2024.27.2.71.

[19] Park H. J. and Seo M. J. , Experience of Healthcare Providers Participating in Life-Sustaining Treatment Decision Making for end-Of-Life Cancer Patient, Journal of the Korea Bioethics Association. (2023) 24, no. 2, 47–68, 10.37305/JKBA.2023.12.24.2.47.

[20] Wu Y. , Mao Y. , Liu Y. et al., Negative Energy Magnetic Field: A Descriptive Qualitative Study on Occupational Stressors Among Chinese Hospice Nurses, Journal of Nursing Management. (2024) 2024, no. 1, 10.1155/2024/3311735. PMC11918853 40224747

[21] Kim Mi J. and Chang H. K. , Factors Affecting Performance of end-Of-Life Care Among ICU Nurses, The Journal of the Convergence on Culture Technology. (2021) 7, no. 3, 135–146, 10.17703/JCCT.2020.7.3.135.

[22] Manz C. C. and Sims H. P.Jr, Leading Self-Managed Groups: A Conceptual Analysis of a Paradox, Economic and Industrial Democracy. (1986) 7, no. 2, 141–165, 10.1177/0143831X867200.

[23] Hwang K. M. , Analysis of Structural Equation Model Between Role Conflict of Clinical Nurses, Positive Psychological Capital, Organizational Effectiveness, and Nursing Performance, The Journal of Learner-Centered Curriculum and Instruction. (2021) 21, no. 6, 509–524, 10.22251/jlcci.2021.21.6.509.

[24] Seligman M. E. P. , Flourish: A Visionary New Understanding of Happiness and Well-Being, 2011, Simon & Schuster, New York.

[25] Kwon S. N. and Park H. J. , Effects of Nurses’ Positive Psychological Capital, Self-Leadership, and Relational Bonds on Organizational Commitment, Journal of Korean Academy of Nursing Administration. (2020) 26, no. 3, 241–250, 10.11111/jkana.2020.26.3.241.

[26] Lee S. N. and Kim J. A. , Concept Analysis of Positive Psychological Capital, Journal of Korean Academy of Nursing Administration. (2017) 23, no. 2, 181–190, 10.11111/jkana.2017.23.2.181.

[27] Morgan Y. , Carolyn M. , and Luthans F. , Psychological Capital and Well-Being, Stress and Health: Journal of the International Society for the Investigation of Stress. (2015) 31, no. 3, 10.1002/smi.2623. 26250352

[28] Lee H. J. , Critical Care Nurses’ Perceived End of Life Care Competencies and Supportive Behaviors and Barriers, 2015, Seoul National University, Master’s Thesis.

[29] Creswell J. W. , A Concise Introduction to Mixed Methods Research, 2015, Sage Publications, Los Angeles.

[30] Creswell J. W. and David Creswell J. , Research Design: Qualitative, Quantitative, and Mixed Methods Approaches, 2017, 4th edition, Sage Publications, Los Angeles.

[31] Montagnini M. , Smith H. , and Balistrieri T. , Assessment of Self-Perceived end-Of-Life Care Competencies of Intensive Care Unit Providers, Journal of Palliative Medicine. (2012) 15, no. 1, 29–36, 10.1089/jpm.2011.0265. 22171962

[32] Jo K. H. , Development and Evaluation of Shared Medical Decision-Making Scale for end-Of-Life Patients in Korea, Journal of Korean Academy of Nursing. (2012) 42, no. 4, 453–465, 10.4040/jkan.2012.42.4.453. 22972206

[33] Kang Bo R. and Oh H. Y. , Development of Ethical Nursing Competence Self-Rating Scale for Clinical Nurses, Korean Journal of Adult Nursing. (2020) 32, no. 5, 482–493, 10.7475/kjan.2020.32.5.482.

[34] Manz C. C. , The Art of Self-Leadership: Strategies for Personal Effectiveness in Your Life and Work, 1983, Prentice-Hall, Englewood Cliffs, NJ.

[35] Kim H. S. , The Relationship Between Teacher’s Self-Leadership and the Jobs Satisfaction at Middle School, 2003, Soongsil University, Master’s Thesis.

[36] Cho K. H. , The Influence of Self-Leadership About Job-Satisfaction and Outcome of Nursing Practice, 2003, Korea University, Master’s Thesis.

[37] Luthans F. , Avolio B. J. , Avey J. B. , and Norman S. M. , Positive Psychological Capital: Measurement and Relationship With Performance and Satisfaction, Personnel Psychology. (2007) 60, no. 3, 541–572, 10.1111/j.1744-6570.2007.00083.x.

[38] Holloway I. and Galvin K. , Qualitative Research in Nursing and Healthcare, 2023, 5th edition, John Wiley & Sons.

[39] Krueger R. A. and Casey M. A. , Focus Groups: A Practical Guide for Applied Research, 2014, Sage Publications, Los Angeles.

[40] Morgan D. L. , Focus Groups as Qualitative Research, 1997, Sage Publications, Thousand Oaks, CA.

[41] Guest G. , Namey E. , and Mckenna K. , How Many Focus Groups Are Enough? Building an Evidence Base for Nonprobability Sample Sizes, Field Methods. (2017) 29, no. 1, 3–22, 10.1177/1525822X16639015.

[42] Braun V. and Clarke V. , Using Thematic Analysis in Psychology, Qualitative Research in Psychology. (2006) 3, no. 2, 77–101, 10.1191/1478088706qp063oa.

[43] Guba E. G. and Lincoln Y. S. , Fourth Generation Evaluation, 1989, Sage Publications, Newbury Park, CA.

[44] Ko E. J. , Lowie S. , and Ni P. , Confidence in Carrying out Palliative Care Among Intensive Care Nurses, Nursing in Critical Care. (2023) 28, no. 1, 13–20, 10.1111/nicc.12735. 34889484

[45] Park E. J. and Seo M. J. , The Influence of Death Anxiety and Terminal Care Stress on Job Satisfaction of New Nurses, Korean Journal of Occupational Health Nursing. (2019) 28, no. 4, 230–241, 10.5807/kjohn.2019.28.4.230.

[46] Abu H. , Bassam W. , and Sperling D. , Views, Attitudes, and Reported Practices of Nephrology Nurses Regarding Shared Decision-Making in end-Of-Life Care, Nursing Ethics. (2024) 31, no. 5, 739–758, 10.1177/09697330231200565. 37794561 PMC11370162

[47] Yun J. H. , Seong Mi H. , Cho Y. M. , and Sok S. H. , Influences of Nursing Professionalism, Empathy, and Clinical Decision-Making Ability on Shared Decision-Making Awareness Among Hemodialysis Nurses, Journal of Nursing Management. (2024) 2024, 2518065–2518069, 10.1155/2024/2518065. 40224815 PMC11919043

[48] Arends S. A. M. , Thodé M. , Pasman H. R. W. , Francke A. L. , and Jongerden I. P. , How Physicians See Nurses’ Role in Decision-Making About Life-Prolonging Treatments in Patients With a Short Life Expectancy: An Interview Study, Patient Education and Counseling. (2023) 114, 10.1016/j.pec.2023.107863. 37356117

[49] Sánchez A. R. , María Jesús Martínez Beltrán , Marín J. M. A. , Julio C. , Gil B. B. , García MdC. M. , and Ribeiro A. S. F. , The Communication of Bad News in Palliative Care: The View of Professionals in Spain, American Journal of Hospice and Palliative Medicine®. (2024) 41, no. 1, 26–37, 10.1177/10499091231163323. 36943176

[50] Seo N. R. and Yeom H. E. , Factors Affecting Psychological Burnout in Nurses Caring for Terminal Cancer Patients, Journal of Hospice and Palliative Care. (2022) 25, no. 4, 159–168, 10.14475/jhpc.2022.25.4.159. 37674666 PMC10179995

[51] Chen F. , Zang Y. , Dong H. , Wang X. , Bian J. , and Lin X. , Effects of a Hospital-Based Leisure Activities Programme on Nurses’ Stress, Self-Perceived Anxiety and Depression: A Mixed Methods Study, Journal of Nursing Management. (2022) 30, no. 1, 243–251, 10.1111/jonm.13484. 34590366 PMC9293447

